# Decoding prognostic factors in SARS-CoV-2 complications among patients with hematological disorders

**DOI:** 10.1016/j.clinsp.2025.100625

**Published:** 2025-03-25

**Authors:** Fengbo Jin, Wei Qian, Yingying Chen, Wanlu Tian, Ling Ge, Mingzhen Yang, Leiming Xia

**Affiliations:** a Department of Hematology, The First Affiliated Hospital of Anhui Medical University, Hefei, China; b Anhui Public Health Clinical Center, Hefei, China; c School of Basic Research, Xinjiang Second Medical College, Xinjiang, China

**Keywords:** SARS-CoV-2, Hematological disorders, Lymphocyte counts, Ferritin, D-dimer, FDP, SARS-CoV-2 pneumonia, Disease Severity, Mortality

## Abstract

Within the intricate tapestry of the global SARS-CoV-2 pandemic, this study delves into the intricate interplay of clinical data to elucidate prognostic factors associated with complications in patients concomitantly afflicted with hematological disorders and SARS-CoV-2. An exhaustive analysis of 71 individuals, spanning the period from November 2022 to March 2023, aims to unveil distinctive clinical characteristics and explicate the nuanced determinants steering the trajectory of the disease. The updated findings reveal a multi-faceted correlation, underscoring the complex interplay of clinical parameters. Among individuals with hematological disorders, anomalously elevated ferritin levels are closely associated with the development of SARS-CoV-2 pneumonia, while interferon-γ is intricately linked to the severity of SARS-CoV-2. Conversely, elevated ferritin levels, increased D-dimer and fibrin degradation products, along with significantly elevated iron levels, manifest a significant association with patient mortality. Intriguingly, those in patients in complete hematologic remission confront an augmented risk of developing SARS-CoV-2 pneumonia, while those abstaining from anti-tumor treatments exhibit mitigated case severity. This study unveils the intricate interplay of clinical factors impacting the prognosis of SARS-CoV-2 complications in individuals with hematological disorders. The cognizance of aberrant interferon-γ activation and nuanced associations with ferritin, iron levels, and coagulation markers contributes to a more holistic comprehension of the prognostic landscape.

## Highlights

• This study addresses the critical prognostic factors for SARS-CoV-2 complications in hematological patients, a high-risk population with reported mortality rates up to 31 %.
• Through a retrospective analysis of 71 hematology inpatients (November 2022–March 2023), elevated ferritin levels emerged as a robust predictor of COVID-19 pneumonia development (*p* = 0.016), while heightened serum interferon-γ concentrations correlated strongly with disease severity (*p* = 0.048).
• Mortality risk was significantly associated with elevated neutrophil counts, ferritin, D-dimer, and fibrin degradation products (*p* < 0.05). Notably, patients with myeloid malignancies in complete remission exhibited increased pneumonia susceptibility (*p* = 0.012), suggesting post-treatment immune vulnerability.
• Conversely, B-cell-targeted therapy or cytotoxic chemotherapy markedly reduced severe infection rates (*p* = 0.024).
• These findings fill critical gaps in prognostic assessment for hematological patients with SARS-CoV-2 co-infection and establish a scientific foundation for personalized monitoring through multidimensional biomarker analysis (e.g., iron metabolism, coagulation profiles, cytokine dynamics), directly informing optimized management strategies for high-risk populations.CLINICS-D-23–00783_Original Article.

## Introduction

The global impact of the SARS-CoV-2 pandemic, driven by the Severe Acute Respiratory Syndrome Coronavirus-2 (SARS-CoV-2), has been profound, with over 273 million confirmed cases and 5.3 million deaths reported worldwide as of December 19th, 2021.^1^^,^^2^ Notably, some individuals initially exhibit no noticeable symptoms upon contracting the virus. However, its progression often leads to severe pneumonia, respiratory distress, organ failure, and tragic fatalities.^3^ The outbreak and spread of this pandemic highlight the substantial threat posed by SARS-CoV-2, presenting unprecedented challenges to global public health.

While scientific advancements have been made in comprehending SARS-CoV-2, prognostic factors for SARS-CoV-2 infections remain incompletely elucidated. Existing studies indicate that age, gender, and underlying health conditions crucially influence the prognosis of SARS-CoV-2 patients.^4^, ^5^, ^6^ Especially, elderly patients with preexisting cardiovascular diseases, hypertension, diabetes, and other underlying conditions, as well as male patients, appear more susceptible to severe cases and a higher risk of mortality.^7^, ^8^, ^9^ Furthermore, several laboratory indicators, such as blood cell counts, biochemical parameters, and coagulation markers, have shown associations with the severity of SARS-CoV-2.^10^, ^11^, ^12^

The threat of SARS-CoV-2 is accentuated for individuals with hematological disorders. Data from the European Hematology Association's Infection Working Group indicate that the mortality rate for adult patients with hematological malignancies who contract the virus can be as high as 31 % within this specific group.^13^ This highlights a heightened risk for severe SARS-CoV-2 outcomes in patients with hematological disorders, potentially linked to immunosuppression induced by their underlying condition or the treatment.^13^, ^14^, ^15^ However, scientific research on how SARS-CoV-2 spreads and affects individuals with hematological disorders remains limited. The complexity of this issue presents significant challenges for related research, emphasizing the urgent need for further investigations to clarify the manifestations and risk factors for SARS-CoV-2 infections in patients with hematological disorders.

In this study, the focus is a thorough examination of 71 patients afflicted with hematological disorders and concurrent SARS-CoV-2 infection. The primary objective was to gain a profound understanding of the distinctive medical requirements and risks associated with this cohort during the SARS-CoV-2 pandemic. We conducted an exhaustive analysis of pertinent prognostic factors, with the intention of elucidating potential outcomes for individuals with hematological disorders post-SARS-CoV-2 infection. Through the meticulous research on this unique patient population, we aspire to establish a robust scientific foundation for the development of more efficacious preventive strategies and treatment modalities.

## Materials and methods

### General information

In accordance with the guidelines issued by the National Health Commission and the National Administration of Traditional Chinese Medicine in the “Diagnosis and Treatment Protocol for SARS-CoV-2 Pneumonia (Trial Version 7)”,^16^ we conducted a study from November 2022 to March 2023 in the Hematology Department. A cohort of 71 patients, all afflicted with hematological disorders and concurrently diagnosed with SARS-CoV-2 meeting the stipulated criteria, was meticulously enrolled. The confirmation of positive cases was established through either pharyngeal swab nucleic acid testing or nasal swab antigen testing. This study has been approved by the Ethics Review Committee of the First Affiliated Hospital of Anhui Medical University (Ethics approval number: PJ-YX2024–048), and patients included in the study are exempt from signing the informed consent due to its observational nature. As a single-arm observational study, we strictly adhere to the rules of the STROBE statement to refine the manuscript.

### Diagnosis and classification criteria

All patients met the diagnostic criteria outlined in the Diagnosis and Treatment Protocol for SARS-CoV-2 Pneumonia (Trial Version 7). Confirmation of the diagnosis relied on a positive result in real-time fluorescent Reverse Transcription-Polymerase Chain Reaction (RT-PCR) testing for SARS-CoV-2 nucleic acid. Clinical classifications upon admission were as follows: (1) Mild cases exhibited mild clinical symptoms with no radiological evidence of pneumonia. (2) Common cases presented with fever, respiratory symptoms, and radiological evidence of pneumonia. (3) Severe cases met any of the following criteria: dyspnea, respiratory rate ≥30 breaths/min, resting oxygen saturation ≤93 %, or the ratio of arterial oxygen partial pressure (PaO_2_) to the fraction of inspired oxygen (FiO_2_) ≤300 mmHg. (4) Critical cases met any of the following criteria: respiratory failure requiring mechanical ventilation, shock, or multi-organ failure necessitating Intensive Care Unit (ICU) management.

### Medical history and data collection

Comprehensive laboratory test results, including complete blood counts, liver and kidney function, coagulation profiles, cytokine levels, ferritin, T-cell subpopulations, and chest CT scan findings, were collected for each patient. Medical history data encompassed disease status, treatment modalities, disease outcomes, and antiviral regimens.

### Statistical methods

We utilized a comprehensive array of statistical methods for data analysis. Descriptive statistics, including means ± standard Error, and percentages, were employed to summarize the data. To assess differences among different groups, *t*-tests, and analysis of variance (ANOVA) were applied. Statistically significant differences were defined as those with a significance level of *p* < 0.05. Statistical analyses were performed using Prism 9 software, ensuring the robustness and reliability of the present results.

## Results

### Patient characteristics

This study encompassed 71 patients, including 42 males and 29 females, with a median age of 57.6-years (range: 22‒86 years). Patients were categorized into different disease types, including 8 cases of benign hematologic disorders (11.3 %), 35 cases of hematologic malignancies of lymphoid origin (49.3 %), and 28 cases of hematologic malignancies of myeloid origin (39.4 %). In terms of treatment, 13 patients received B-cell targeted therapy (18.3 %), 39 patients received cytotoxic drug therapy (54.9 %), 17 patients did not receive primary disease treatment (23.9 %), and one patient underwent hematopoietic stem cell transplantation and hormone shock therapy (the therapy defined by high-dose dexamethasone of 40 mg/day administered intravenously), respectively. Among the 63 patients with hematologic malignancies, 14 patients achieved complete remission of the primary disease, accounting for 22.2 % of the total. Regarding SARS-CoV-2 pneumonia, 53 patients were involved (74.6 %), with 20 severe cases (37.7 %). During inpatient treatment, 38 patients received antibacterial treatment in addition to antiviral therapy (71.7 %), and 29 patients concurrently received antifungal treatment (54.7 %). Eight unfortunate deaths occurred during the observation period, constituting 11.3 % of the total. Further details are presented in Table 1.

**Table 1:** Patient characteristics.

Characteristics	Cases (Percentage, %)
Sex	71
Female	29 (40.85 %)
Male	42 (59.15 %)
Age, Median (range)	57.59 (22 to 86)
Duration of COVID Infection (days), Mean±SD	13.86±8.36
Disease Classification	
*Lymphocytes and Plasma Cell Tumors*	35 (49.30 %)
Acute Lymphoblastic Leukemia	3 (8.57 %)
Monoclonal Gammopathy of Undetermined Significance	1 (2.86 %)
Multiple Myeloma	12 (34.29 %)
Diffuse Large B-Cell Lymphoma	4 (11.43 %)
Large Granular Lymphocytic Leukemia	1 (2.86 %)
Mantle Cell Lymphoma	5 (14.29 %)
Angioimmunoblastic T-Cell Lymphoma	1 (2.86 %)
Lymphoma (other)	4 (11.43 %)
Chronic Lymphocytic Leukemia	5 (14.29 %)
*Myeloid Neoplasms*	28 (39.44 %)
Acute Myeloid Leukemia	15 (53.57 %)
Acute Promyelocytic Leukemia	1 (3.57 %)
Chronic Myeloid Leukemia	1 (3.57 %)
Myelodysplastic Syndromes	7 (25.00 %)
Primary Myelofibrosis	1 (3.57 %)
Mixed Phenotype Acute Leukemia	1 (3.57 %)
Chronic Myelomonocytic Leukemia	1 (3.57 %)
*Non-Malignant Hematologic Disorders*	8 (11.27 %)
Aplastic Anemia	2 (25.00 %)
Immune Thrombocytopenia	3 (37.50 %)
Paroxysmal Nocturnal Hemoglobinuria	1 (12.50 %)
Pancytopenia	1 (12.50 %)
Other	1 (12.50 %)
Disease Remission Status	63 (88.73 %)
CR	14/63 (22.22 %)
Non-CR	49/63 (77.78 %)
Receiving Chemotherapy (Yes/No)	71 (100.00 %)
B cell targeting^a^	13 (18.31 %)
Cytotoxicity	39 (54.93 %)
No	17 (23.94 %)
Not include	2^b^ (2.82 %)
History of Chemotherapy in the Last 3-Months	
Coronavirus Pneumonia (Yes/No)	71 (100.00 %)
Yes	53 (74.65 %)
No	18 (25.35 %)
Undergoing Antimicrobial Treatment	67 (94.37 %)
Antibacterial Treatment	38 (56.72 %)
Antifungal Treatment	29 (43.28 %)
Severe (Yes/No)	71 (100.00 %)
Yes	20 (28.17 %)
No	51 (71.83 %)
Deceased (Yes/No)	71 (100.00 %)
Yes	8 (11.27 %)
No	63 (88.73 %)

a Three patients received BTK inhibitor treatment, while ten received treatment with Merohem.

b Two cases, one receiving bone marrow transplantation and the other undergoing hormone therapy, are reported.

### Comprehensive analysis of clinical data associated with patients of hematology department based on SARS-CoV-2 pneumonia development, disease severity, and mortality

In evaluating the risk factors among hematology department inpatients with SARS-CoV-2 pneumonia (NCP), the authors conducted a comprehensive analysis based on three specific observational targets: the development of NCP, disease severity, and mortality. Initially, when patients were grouped according to the development of NCP, the authors observed a significant difference in Ferritin expression between the two groups, with a p-value of 0.0160, indicating that NCP patients had significantly higher Ferritin levels than non-NCP patients. Subsequently, upon categorizing patients based on the severity of the disease, a statistically significant difference in serum Interferon-γ concentration was observed, with a p-value of 0.0484. The concentration of serum Interferon-γ (31.72 ± 12.18) was significantly elevated in severe patients compared to non-severe patients (10.75 ± 3.466). In the final analysis, patients were grouped by mortality, revealing four risk factors ‒ Neutrophil count, Ferritin, d-Dimer, and Fibrin Degradation Products ‒ with statistically significant differences between the two groups. It is noteworthy that the Neutrophil count (7.678 ± 3.153) in deceased patients was significantly higher than in non-deceased patients (6.737 ± 4.426). Additionally, Ferritin (3487 ± 949.8) in deceased patients was significantly higher than in non-deceased patients (1333 ± 296.6). d-Dimer (4.101 ± 1.425) showed a marked elevation in deceased patients compared to non-deceased patients (0.8967 ± 0.2924), and Fibrin Degradation Products (12.34 ± 10.32) were also significantly elevated in deceased patients compared to non-deceased patients (2.666 ± 0.6102). Detailed results are presented in Table 2.

**Table 2:** Comprehensive Analysis of Clinical Data Associated with Patients of Hematology Department Based on Novel Coronavirus Pneumonia Development, Disease Severity, and Mortality.

Comprehensive Analysis of Clinical Data Associated with Patients of Hematology Department Based on Novel Coronavirus Pneumonia Development, Disease Severity, and Mortality.										
		**Whether Coronavirus Pneumonia Occurred**			**Whether Severe Case**			**Whether Deceased**		
		**Coronavirus Pneumonia (N)**	**No Coronavirus Pneumonia (N)**	**p-value**	**Severe Cases (N)**	**Non-severe Cases (N)**	**p-value**	**Deceased (N)**	**No Deceased (N)**	**p-value**
Age	≥ 60-years old	30	11	0.7888	8	33	0.0678	2	39	0.0631
	< 60-years old	23	7		12	18		6	24	
Gender	Male	34	8	0.1715	14	28	0.2917	5	37	>0.9999
	Female	19	10		6	23		3	26	
White Blood Cells (× 10^9^/L)		11.43±5.01 (53)	24.05±16.96 (18)	0.8212	24.69±12.92 (20)	10.69±6.05 (51)	0.2692	33.82±23.91 (8)	12.19±5.64 (63)	0.2297
Neutrophils (× 10^9^/L)		2.99±0.572 (53)	18.20±15.45 (18)	0.9446	4.51±1.39 (20)	7.76±5.47 (51)	0.1267	7.68±3.15 (8)	6.74±4.43 (63)	0.0226
Lymphocytes (× 10^9^/L)		8.04±4.82 (53)	2.68±1.29 (16)	0.9295	19.69±12.51 (20)	1.48±0.42 (51)	0.3016	25.09±23.56 (8)	4.260±2.80 (63)	0.5463
Hemoglobin (g/L)		86.49±3.71 (53)	84.47±6.93 (17)	0.7922	85.45±6.55 (20)	86.22±3.77 (50)	0.9158	88.38±12.77 (8)	85.69±3.33 (62)	0.7953
Platelets (× 10^9^/L)		108.70±11.01 (53)	192.40±67.94 (17)	0.4088	135.00±20.44 (20)	126.60±24.91 (50)	0.2428	115.50±31.50 (8)	130.80±20.71 (62)	0.7968
CD3^+^*T* Lymphocytes (/μL)		533.00±78.95 (27)	279.20±90.93 (7)	0.1292	543.70±107.90 (12)	446.40±86.69 (22)	0.4983	580.20±216.70 (5)	463.60±70.92 (29)	0.5477
CD4^+^*T* Lymphocytes (/μL)		199.20±36.44 (27)	139.20±55.33 (7)	0.4427	169.30±50.44 (13)	197.70±40.15 (21)	0.663	113.00±23.40 (5)	199.60±35.75 (29)	0.5117
CD8^+^*T* Lymphocytes (/μL)		261.60±56.17 (27)	114.50±32.45 (7)	0.3531	290.50±89.64 (12)	199.10±52.10 (22)	0.3507	409.40±200.40 (5)	200.60±41.38 (29)	0.2754
Regulatory T Lymphocytes (/μL)		15.35±6.78 (20)	7.22±3.70 (4)	0.6052	16.07±11.07 (11)	12.25±5.23 (13)	0.7459	5.74±2.63 (5)	16.17±7.11 (19)	0.785
Albumin (g/L)		33.07±0.90 (51)	34.74±0.95 (18)	0.1495	32.24±1.16 (20)	34.03±0.88 (49)	0.2542	30.23±1.69 (8)	33.94±0.76 (61)	0.0938
Globulin (g/L)		26.32±1.14 (51)	24.91±1.49 (18)	0.5085	27.30±1.32 (20)	25.40±1.18 (49)	0.3572	27.51±1.17 (8)	25.75±1.04 (61)	0.2331
Ferritin (g/L)		1889.00±382.90 (20)	667.00±262.70 (7)	0.016	2620.00±681.40 (6)	1273.00±323.50 (21)	0.0675	3487.00±949.80 (3)	1333.00±296.60 (24)	0.0245
Fibrinogen(g/L)		3.73±0.21 (51)	3.50±0.41 (16)	0.616	3.52±0.33 (20)	3.74±0.23 (47)	0.6094	3.03±0.44 (8)	3.76±0.21 (59)	0.2129
D-Dimer (μg/L)		1.13±0.28 (51)	1.86±1.17 (14)	0.7373	2.04±0.67 (20)	0.96±0.37 (45)	0.1324	4.10±1.43 (8)	0.90±0.29 (57)	0.0003
Fibrin Degradation Products (μg/mL)		3.63±1.49 (14)	5.63±4.19 (2)	0.5723	4.95±2.55 (8)	2.52±0.86 (10)	0.1392	12.34±10.32 (2)	2.67±0.61 (14)	0.0067
Interleukin-6 (pg/mL)		164.70±63.38 (31)	206.30±112.80 (4)	0.8211	259.60±125.60 (15)	101.80±31.24 (20)	0.529	331.50±247.50 (6)	135.90±47.94 (29)	0.1286
Interleukin-10 (pg/mL)		8.96±3.48 (28)	13.70±12.30 (3)	0.6786	7.58±2.36 (13)	10.75±5.47 (18)	0.5404	10.78±4.53 (5)	9.16±3.86 (26)	0.8606
Interferon-γ (pg/mL)		19.53±6.20 (28)	14.07±8.04 (8)	0.6633	31.72±12.18 (13)	10.75±3.47 (18)	0.0484	34.50±19.75 (5)	15.71±5.03 (31)	0.2079

### The relationship between primary disease remission and NCP, severity, and mortality in malignant hematologic patients with SARS-CoV-2 infection

In addition to assessing the correlation between various clinical data and the progression, severity, and mortality of patients infected with the NCP, the authors further grouped patients with malignant diseases of the myeloid and lymphoid hematopoietic systems based on whether the primary disease achieved remission. The authors analyzed inter-group differences in the occurrence of NCP, disease severity, and mortality, as outlined in Table 3. Interestingly, the authors found that, irrespective of the resolution of the primary disease, severe cases and fatalities did not significantly differ among patients with malignant diseases of the myeloid and lymphoid hematopoietic systems after contracting the SARS-CoV-2. However, when grouping patients based on the occurrence of SARS-CoV-2 pneumonia, the authors observed that, regardless of disease improvement, there was no significant difference in the occurrence of NCP among patients with malignant diseases of the lymphoid hematopoietic system. In contrast, patients with malignant diseases of the myeloid hematopoietic system, especially those whose primary disease completely resolved, exhibited a higher risk of developing SARS-CoV-2 pneumonia, with statistical significance (p-value of 0.012), as detailed in Table 3.

**Table 3:** The relationship between primary disease remission and NCP, severity, and mortality in malignant hematologic patients with COVID-19 infection.

		Whether Coronavirus Pneumonia Occurred			Whether Severe Case			Whether Deceased		
		Coronavirus Pneumonia (N)	No Coronavirus Pneumonia (N)	p-value	Severe Cases (N)	Non-severe Cases (N)	p-value	Deceased (N)	No Deceased (N)	p-value
Myeloid Disorders	CR	4	0	0,012	2	2	0,5583	0	3	>0.9999
	Non-CR	6	17		6	17		1	23	
Lymphoplasmacytic Disorders	CR	10	2	0,6855	1	11	0,1149	1	11	0,6552
	Non-CR	17	7		9	15		5	25	

### Exploring treatment modalities and SARS-CoV-2 outcomes in malignant hematologic patients

**Fig. 1:**
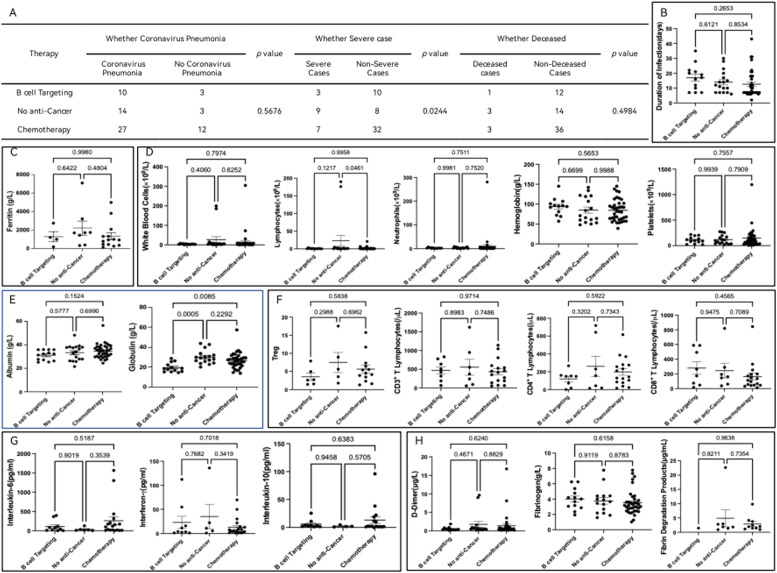
Impact of Treatment Modalities on the Severity of COVID-19 in Malignant Hematologic Patients. (A) Patients in the B-cell targeting group and the chemotherapy group showed a significantly reduced proportion of severe SARS-CoV-2 cases compared to the No anti-Cancer group, with a p-value of 0.0244. (B) Despite a decrease in the proportion of severe cases with B-cell targeting and chemotherapy, there was no significant difference in the duration of hospitalization among the B-cell targeting, chemotherapy, and No anti-Cancer groups. (C‒H) Further, the authors evaluated the differences in Biomarkers among the three groups. (C) No statistically significant differences were found in serum ferritin concentrations across the B-cell targeting, chemotherapy, and No anti-Cancer groups. (D) No statistically significant differences in peripheral blood white blood cell, lymphocyte, neutrophil, hemoglobin, and platelet counts were observed among the three groups. (E) For serum albumin and globulin comparison, albumin levels were not statistically different among the groups. However, globulin levels were significantly lower in the B-cell targeting group compared to the chemotherapy and No anti-Cancer groups, with no significant difference between the chemotherapy and No anti-Cancer groups. (F) Regarding T-cell subset distribution, no statistically significant differences were noted in the proportion of Treg and the absolute counts of CD3, CD4, and CD8 T-lymphocytes among the three groups. (G) There were no statistically significant differences in interleukin-6, interferon-gamma, and interleukin-10 levels among the B-cell targeting, chemotherapy, and No anti-Cancer groups. (H) No statistically significant differences were found in D-dimer, fibrinogen, and fibrin degradation product levels among the three groups.

In light of the observation that complete remission of myeloid diseases may be linked to SARS-CoV-2 pneumonia, possibly through complex mechanisms associated with enhanced antiviral immune capabilities in these patients, the earlier findings also indicated elevated lymphocyte counts and interferon-gamma levels in critical cases of malignant hematologic inpatients with SARS-CoV-2. Motivated by these insights, the authors delved into an investigation to ascertain whether different treatment approaches were correlated with SARS-CoV-2 pneumonia, severity, and mortality. This exploration is especially pertinent due to the significant impact that B-cell-targeted therapy and cytotoxic treatments can have on patients' immune functions in hematologic care. The investigation revealed a noteworthy finding: patients undergoing B-cell-targeted therapy and cytotoxic chemotherapy exhibited a substantially lower proportion of severe SARS-CoV-2 cases compared to those not undergoing anti-tumor treatment, with a p-value of 0.0244 (refer to Fig. 1A). However, when considering the overall perspective, there was no significant difference in the duration of hospitalization among patients receiving B-cell-targeted therapy, cytotoxic treatment, and those not undergoing anti-tumor therapy (see Fig. 1B). Furthermore, the comprehensive analysis encompassing ferritin (Fig. 1C), key parameters in blood routine tests (Fig. 1D), albumin, globulin (Fig. 1E), T-cell subsets (Fig. 1F), cytokines (Fig. 1G), and major coagulation indicators (Fig. 1H) unveiled a statistically significant decrease in globulin levels in the B-cell-targeted therapy group compared to the other two groups (refer to Fig. 1E).

## Discussion

Despite the temporary easing of the global SARS-CoV-2 pandemic, ongoing analysis of the infection remains critical for enhancing the comprehension of the virus's nature. The present findings highlight three pivotal points: First, individuals with SARS-CoV-2 pneumonia have markedly higher ferritin levels, and those with severe cases exhibit increased serum interferon-gamma levels, with deceased patients showing significantly higher levels of neutrophil count, ferritin, d-dimer, and fibrin degradation products compared to those who survived. Second, there's no significant variation in the occurrence of severe cases and mortality rates among patients with myeloid and lymphoid hematologic malignancies, although patients with myeloid malignancies in complete remission are at a higher risk for SARS-CoV-2 pneumonia. Third, the proportion of severe cases is significantly reduced in patients receiving B-cell targeted therapy and cytotoxic chemotherapy compared to those untreated, with no discernible difference in hospital stay among the groups, and a pronounced decrease in globulin levels observed in the B-cell targeted therapy group. These insights provide a clearer view of the SARS-CoV-2 impact on patients with hematologic diseases and the effects of various treatments on disease progression, offering direction for future research and clinical approaches.

In assessing the risk factors for SARS-CoV-2 pneumonia among hospitalized hematology patients, the authors observed that patients with pneumonia had significantly higher ferritin levels than those without pneumonia, and patients with severe infections had significantly higher serum Interferon-Gamma (IFN-γ) concentrations than those with mild infections. Additionally, deceased patients had markedly higher levels of neutrophil count, ferritin, D-dimer, and Fibrin Degradation Products (FDP) compared to survivors. Currently, the correlation between elevated ferritin and SARS-CoV-2 pneumonia is primarily manifested in the following aspects: First, as an inflammatory marker, the increase in ferritin levels is commonly regarded as an indicator of inflammation. In SARS-CoV-2 patients, the activation of the immune system may lead to inflammatory responses, resulting in elevated ferritin levels.^17^^,^^18^ Second, in terms of immune response, ferritin is involved in regulating the body's iron metabolism, which is crucial for the normal functioning of the immune system. Therefore, the abnormal increase in ferritin levels may reflect an overactivation or dysregulation of the immune system, which is to some extent associated with the immune response to SARS-CoV-2.^17^^,^^19^ Lastly, regarding tissue damage, the SARS-CoV-2 virus can cause damage to the lungs and other tissues. Tissue damage may lead to the release of ferritin, and consequently, elevated ferritin levels may reflect the extent of tissue damage during the disease.^20^ Furthermore, this study emphasizes the close association between elevated IFN-γ levels and the severity of the disease. In the field of SARS-CoV-2 pathophysiology, some studies suggest a potential link between sphingolipid metabolism and lymphocyte release, proposing a mechanism wherein the conversion of sphingosine to sphingosine-1-phosphate may contribute to systemic inflammation and the subsequent release of cytokines and chemokines.^17^ In addition, the role of IFN-γ in SARS-CoV-2 is critical as a cytokine central to immune response regulation and antiviral activities.^18^ Contradictory research results surround its involvement in immune dysregulation and conditions such as pulmonary fibrosis. While early IFN treatment emerges as a potential mitigator of infection severity, an individual's genetic response to IFN-γ may intricately tie with susceptibility. Remarkably, in asymptomatic infection, heightened IFN-γ levels correlate with robust antiviral immune responses without inducing apparent pathology.^17^ In summary, the present study results confirm that patients with pneumonia have significantly higher ferritin levels than those without pneumonia, and patients with severe infections have significantly higher serum IFN-γ concentrations than those with mild infections, consistent with existing knowledge. In this study, the authors conducted a comprehensive analysis of factors closely linked to patient mortality in the context of SARS-CoV-2. Notably, decreased ferritin levels, elevated D-dimer concentrations, and increased FDP were identified as statistically significant contributors to adverse outcomes.^20^ These findings align with previous research, reinforcing the importance of these biomarkers in assessing the severity and prognosis of SARS-CoV-2 cases. The retrospective observational study included 318 SARS-CoV-2 patients with a survival rate of 78.6 %. The authors found that survivors had an average D-dimer of 2.1 mg/mL and an average ferritin level of 698.59 ng/mL. In contrast, non-recovered patients exhibited higher levels of D-dimer (5.46 mg/mL) and ferritin (992.96 ng/mL).^19^ These findings underscore the potential utility of ferritin and D-dimer as prognostic indicators for the severity and mortality of SARS-CoV-2 infection. Moreover, another study corroborates the significance of elevated D-dimer levels in predicting the mortality risk among SARS-CoV-2 patients.^9^ The present results contribute to the growing body of evidence emphasizing the crucial role of these hematologic markers in understanding and managing outcomes of SARS-CoV-2.

Although a definitive association between types of hematologic diseases and SARS-CoV-2 infection remains uncertain, in the analysis of the clinical data of patients with SARS-CoV-2 pneumonia in relation to disease progression, severity, and mortality, the authors found no significant difference in severe cases and mortality rates between patients with myeloid and lymphoid hematologic malignancies. However, patients with myeloid hematologic malignancies who achieved complete remission of their primary disease showed an increased risk of developing SARS-CoV-2 pneumonia, while there was no significant difference in the risk of SARS-CoV-2 pneumonia occurrence among patients with lymphoid hematologic malignancies. Here, the results differ from current reports, where lymphoproliferative diseases, particularly non-Hodgkin lymphoma and multiple myeloma, may predispose individuals to SARS-CoV-2 infection. Additionally, the literature indicates a higher incidence of SARS-CoV-2 infection among patients with acute myeloid leukemia.^13^^,^^21^^,^^22^ The present study's data reveal that among individuals infected with SARS-CoV-2, 39.44 % (28/71) had acute myeloid leukemia, and 49.30 % (35/71) had lymphoproliferative diseases of the lymphoplasmacytic system. Due to data limitations, this may not accurately reflect the distribution of hematologic diseases infected locally. However, lymphoproliferative diseases of the lymphoplasmacytic system still account for a considerable proportion. Furthermore, the study found no significant correlation between disease type and the occurrence, severity, or mortality of SARS-CoV-2 pneumonia.

Current data indicate that the severity of SARS-CoV-2 infection in hosts depends on two main factors: the overall health status of the host and the virulence of SARS-CoV-2. Severe cases of SARS-CoV-2 are closely associated with age (> 65-years) and underlying conditions such as diabetes, hypertension, and chronic pulmonary diseases.^4^, ^5^, ^6^, ^7^, ^8^, ^9^ It is noteworthy that some individuals infected with SARS-CoV-2 initially present mild symptoms but can rapidly deteriorate, leading to severe pneumonia and ultimately multiple organ failure, primarily due to a “cytokine storm”.^23^, ^24^, ^25^ Specific groups of cancer patients, particularly those with hematologic malignancies and recipients of hematopoietic stem cell transplantation, have been reported to have a higher incidence of SARS-CoV-2 infection and are more prone to developing severe pneumonia.^26^, ^27^, ^28^ Hematologic disease patients generally exhibit various potential risk factors, including aging, frequent coexistence of immune dysfunction, abnormal composition of immunocytes, and the impact of treatments such as chemotherapy, targeted therapy, and oral immunosuppressive agents. These factors result in concurrent bone marrow suppression and immune system suppression, often leading to clinical manifestations in patients admitted to hematology departments, such as neutropenia, lymphocytopenia, anemia, thrombocytopenia, T-cell subset imbalance, and immunoglobulin deficiency, all closely associated with SARS-CoV-2 infection and its progression.

In this study, these observations underscore a heightened risk of SARS-CoV-2 pneumonia among patients with hematologic malignancies, especially those in complete remission of their primary disease. Conversely, individuals who have not undergone anti-tumor therapy during the SARS-CoV-2 infection period show a significantly lower proportion of severe cases. As suggested in recent literature,^20^ various risk factors influence the severity of SARS-CoV-2 infection in cancer patients, including delayed hospital admission, low sensitivity, or misinterpretation of SARS-CoV-2 RT-PCR tests. The similarity of cancer symptoms to SARS-CoV-2 can lead to initial misdiagnosis in some cases. Additionally, treatment-related risk factors for cancer patients encompass chemotherapy, targeted therapy, radiotherapy, immunotherapy, and treatment regimens containing JAKi or BTKi. High-dose corticosteroids and Immune Checkpoint Inhibitors (ICIs) are also implicated in increasing the risk of infection. Patchy consolidation observed in the initial CT scan of the lungs upon admission serves as a risk factor for increased infection severity. Furthermore, susceptibility to infection varies among patients with different types of cancers. Overall, within the hematology department, patients with malignancies requiring primary disease treatment exhibit a markedly increased risk of severe progression, while those in remission or undergoing elective treatment for the primary disease may be relatively less prone to developing SARS-CoV-2 pneumonia. Continued research and understanding of these dynamics are essential for optimizing clinical management and treatment strategies in this context.

## Conclusions

In this retrospective study, the authors systematically analyzed the clinical data of patients within the hematology department who were affected by SARS-CoV-2 infection. The investigation yielded valuable insights, particularly concerning various hematological parameters. These findings offer a valuable perspective on the unique biological features of SARS-CoV-2 infection in individuals with hematological conditions. It is noteworthy that the authors observed a close association between elevated ferritin levels and interferon-γ levels with the occurrence and severity of SARS-CoV-2. Additionally, elevated levels of neutrophils, ferritin, D-dimer, and fibrin degradation products showed a significant correlation with patient mortality. Furthermore, the observations highlighted those individuals diagnosed with malignant hematologic diseases, especially those in complete remission of the primary disease, face an increased risk of contracting SARS-CoV-2 pneumonia. Interestingly, among patients who had not undergone antitumor therapy, the incidence of severe SARS-CoV-2 cases was notably lower. These findings provide important insights for a deeper understanding of the presentation and outcomes of hematological patients affected by SARS-CoV-2 infection.

## Funding

This work was financially supported by the 10.13039/100016073Key Research and Development Program of Anhui Province (Grant number 202104j07020028) and the General Scientific Research Foundation of the Education Department of Anhui (Grant number KJ2021A0333 and KJ2021A0330).

## Declaration of competing interest

The authors declare no conflicts of interest.
